# Inactivation of *Escherichia Coli* and *Salmonella* Using 365 and 395 nm High Intensity Pulsed Light Emitting Diodes

**DOI:** 10.3390/foods8120679

**Published:** 2019-12-13

**Authors:** Amritha Prasad, Michael Gänzle, M. S. Roopesh

**Affiliations:** 1 Department of Agricultural, Food and Nutritional Science, University of Alberta, Edmonton, AB T6G 2R3, Canada; amritha@ualberta.ca (A.P.); mgaenzle@ualberta.ca (M.G.); 2 College of Bioengineering and Food Science, Hubei University of Technology, Wuhan 430086, China

**Keywords:** Light Emitting Diode, *Escherichia coli*, *Salmonella*, antibacterial efficacy, low-a_w_ food

## Abstract

High intensity pulsed light emitting diode (LED) treatment is a novel approach to inactivate foodborne pathogens. The objective of this study was to evaluate the antibacterial potential of high intensity 365 (UV-A) and 395 nm (NUV-Vis) LED treatments against *Escherichia coli* and *Salmonella enterica* at high and low water activity (a_w_) conditions, and to understand the influence of different process parameters on their antibacterial efficacy. Bacteria at high (in phosphate buffer saline, PBS) and low a_w_ (a_w_ = 0.75) conditions were treated with both the LEDs with specific doses at a fixed distance from the LEDs. The 365 nm LED showed more effectiveness in reducing the dried bacteria compared to 395 nm LED. The dry *E. coli* showed more resistance to LED treatments compared to *Salmonella*. The 365 and 395 nm LED treatments with ~658 J/cm^2^ dose resulted in reductions of 0.79 and 1.76 log CFU/g of *Salmonella*, respectively, on 0.75 a_w_ pet foods. The LED treatments increased the surface temperature, resulting in water loss in the treated samples. This study showed that the dose, duration of light exposure, bacterial strain, and a_w_ played a major role in the antibacterial efficacy of the 365 and 395 nm LEDs.

## 1. Introduction

Growth of foodborne pathogenic microorganisms in foods requires a high a_w_. Low-a_w_ foods with a_w_ < 0.85 do not support their growth, but these microorganisms may survive in the dry state throughout the storage life of the foods [1]. Dry foods including pet foods, cereals, and spice powders can cause foodborne illnesses after contamination with *Salmonella enterica* or Shiga-toxin producing *Escherichia coli* [2,3]. Some low-a_w_ foods including nuts were contaminated by *Salmonella enterica* spp. before harvest, while in products such as spray dried milk powder, contamination can occur from product handling or processing environment [2]. A decrease in a_w_ increases the heat resistance of the foodborne pathogens and makes them more resistant to conventional decontamination methods including thermal treatments [4,5]. Pathogen inactivation on dry foods thus requires prolonged thermal treatments, which adversely affect the quality and nutritional composition of the food products. Hence, alternate food decontamination technologies are necessary to achieve elimination of pathogens in dry foods without compromising food quality. Four decontamination technologies for dry foods like almonds were approved by the FDA, which involve the application of propylene oxide, hot oil, hot water, or steam [6].

Pulsed light treatment is an efficient antimicrobial technology, which uses short, intense pulses of light ranging from Ultraviolet (UV) to Near Infrared (NIR) [7]. The antibacterial efficacy of pulsed light has been attributed majorly to the UV spectrum, which is divided into UV-A (320–400 nm), UV-B (280–320 nm), UV-C (200–280 nm) and far UV (100–200 nm) [8,9,10,11]. However, the lamps emitting the pulsed light require long warm-up times and have reduced efficiency at low temperatures [8,9].

Light emitting diodes (LED) emitting monochromatic light are an emerging alternative for decontamination of food. LED technology uses semiconductors that release energy in the form of light with a specific wavelength that depends on the type of semi-conductor material used [9,12]. The LED system does not require warm-up time to start and produce a uniform irradiation [13,14]. Moreover, the longer life span, easy incorporation to the existing processing line owing to its compact design and low voltage requirement, may make it a cost-effective alternative in the future [15,16].

UV-C technology is used for disinfection of air and water, and for sterilization of food surfaces [17,18,19,20]. UV light with lower wavelengths <315 nm (UV-B and UV-C) are hazardous to skin and causes sunburn and mutations, while UV-A is safer to handle [21,22]. UV-A LED treatment is an emerging microbial intervention process for water treatment [23,24]. UV light with wavelengths close to visible region (~395 nm) is called Near UV-Visible (NUV-Vis) light, which has also been proposed as a reliable antimicrobial technology. The 395 nm LED reduced cell counts of *E. coli* O157:H7 in liquid suspension and wheat flour [25,26]. The antibacterial efficacy of the LEDs could be due to the oxidative stress produced in the bacteria. Moreover, visible light could induce photodynamic inactivation in the bacteria [13,27,28].

Although previous studies reported antimicrobial potential of 365 and 395 nm light emitted from LEDs, information about their antimicrobial efficacy at low-a_w_ conditions and for the treatment of foods is limited. Therefore, understanding the antimicrobial efficacy of high intensity pulsed LED system in high and low-a_w_ conditions would help in developing a novel technology for surface sanitation of high and low-a_w_ food products. The main objective of this study was to evaluate the antibacterial effectiveness of 365 and 395 nm LED system against *E. coli* and *Salmonella enterica* spp. in both high and low-a_w_ conditions and to evaluate their efficacy in low-a_w_ foods such as pet foods.

## 2. Materials and Methods

### 2.1. Sample Preparation

*Escherichia coli* AW1.7, a heat resistant food isolate strain encoding the locus of heat resistance [29], *Salmonella enterica* Typhimurium ATCC13311, the heat resistant *S. enterica* Senftenberg ATCC43845 [30], and the waste water isolates *Salmonella enterica* FUA1946, FUA1934, FUA1955 were used in this study. The heat resistant food and wastewater isolates of bacteria were chosen for the study to evaluate whether these strains can survive LED treatments as long-term LED treatments also generate heat. Tryptic soy agar plates (TSA, Becton, Dickinson and Company, Franklin Lakes, NJ, USA) supplemented with 0.6% yeast extract (YE; Fischer Bioreagents, Geel, Belgium) were used to restore the frozen bacterial stock cultures. This was followed by two consecutive transfers in 5 mL sterile tryptic soy broth (TSB; Becton, Dickinson and Company, Franklin Lakes, NJ, USA) supplemented with 0.6% YE and incubation at 37 °C for 18–24 h.

Dried samples of *E. coli* AW1.7 and *S. enterica* ATCC13311 were prepared according to the procedure reported previously with some modifications [31,32]. After restoration of the strains from the stock cultures, 100 µL of culture was spread on TSAYE plates and incubated for 24 h at 37 °C. The bacterial lawn was washed with 1.5 mL of 0.1% peptone water (Fischer Bioreagents, Geel, Belgium) and collected in 1.5 mL Eppendorf tubes followed by centrifugation and another washing step with 1 mL of 0.1% peptone water. The initial cell count of the suspension was 10^12^ CFU/mL. These cell suspensions were then transferred to sterile glass vials (2 mL in each vial) and air dried in a biosafety cabinet for 4–5 days which resulted in 1.56 log reduction in bacterial cell counts, followed by drying over silica gel for 24 h. The dried cells were equilibrated for 7 days to an a_w_ of 0.75 inside an air-tight equilibration chamber containing a supersaturated sodium chloride solution. The a_w_ was confirmed using the water activity meter (4TE, patent number 5816704, Aqualab, Pullman, WA, USA). The final concentration of the cells after equilibration was 10^9^–10^10^ CFU/g resulting in an overall log reduction of 1.82 CFU/mL.

To test the antibacterial efficacy of LED treatments on *Salmonella* inoculated pet foods, dry pet food pellets with initial a_w_ of ~0.54 were purchased from a local store. Each pellet weighed ~0.15 g and was ovular in shape and concave on one side. The pet foods were composed of 44% crude protein, 15% fat, 10% moisture, minerals, and lipids. 15 µL of a five-strain cocktail of *Salmonella enterica* (prepared in a similar manner as mentioned above for the preparation of cell suspension for dry bacteria) was inoculated on the concave side of each pellet and air dried for 45–60 min in the biosafety cabinet to facilitate the attachment of cells on the surface of pet foods, which resulted in 0.95 log (CFU/g) reduction of bacteria, followed by equilibration to 0.75 a_w_ by storing the samples in the equilibration chamber for 3–4 days resulting in an overall cell count reduction by 1.52 log (CFU/g). The final cell count of *Salmonella enterica* in pet foods after equilibration was 10^9^ CFU/g.

### 2.2. Light Emitting Diode (LED) System

The LED system consisted of a bench-top controller (CF3000, Clearstone Technologies Inc., Hopkins, MN, USA) compatible with the JL3 series LED heads (111 × 70 × 128 mm^3^; 6 high intensity LEDs), emitting the light with wavelengths of 365 and 395 nm with an irradiance of 0.05 and 0.23 W/cm^2^ (when the distance was 4 cm from the sample) and 0.114 and 0.55 W/cm^2^ (when the distance was 2 cm from the product), respectively, at 60% power level. The total doses (J/cm^2^) of 365 and 395 nm LEDs during treatments were determined using a laser energy meter (7Z01580, Starbright, Ophir Photonics, A Newport Company, Har Hotzvim, JRS, Israel), connected to a photodiode irradiance and dosage sensor (PD300RM-8W, Ophir Photonics, A Newport Corporation Brand, Har Hotzvim, JRS, Israel). The distance between the LED head and the sensor was maintained at 4 and 2 cm to determine the dose per surface area. The spectra of the 365 and 395 nm LEDs were confirmed using the StellarNet Inc. spectrometer (Black Comet C-25). The frequency of both the LEDs was 100 Hz and the dose values depended on the power level/duty cycle selected. For example, a power level of 60% corresponds to the ‘ON’ and ‘OFF’ times of 6 and 4 ms, respectively. Pet foods were treated at a 2 cm distance and the other samples were treated with a 4 cm distance from the LED head. These treatment heights were selected based on the preliminary experiments. The surface temperature of the samples during the treatments was determined using a thermocouple connected to a digital thermometer (1507726, Fischer Scientific, Hampton, NH, USA). Doses of each LED varied based on the power level output set on the controller system, ranging from 0 to 100%, which is related to the pulse width of the LED treatment (Table 1 and Table 2).

### 2.3. Inactivation of E. coli and Salmonella enterica Cells in Phosphate Buffer Saline

Overnight culture of *E. coli* AW1.7 and *S. enterica* ATCC13311 (1 mL) were mixed with 5 mL phosphate buffered saline, pH 7.4 (PBS; Gibco, life technologies, USA). One mL of this solution was spread in a petri dish with area of 28.3 cm^2^ and treated with the LEDs. The treatments were conducted with 365 and 395 nm LEDs at power levels ranging from 20, 40 and 60% for *E. coli* and 60% for *Salmonella enterica* and treatment times between 10 and 60 min (365 nm) or 5 and 60 min (395 nm). The doses of the treatments ranged from 9.6 to 188.07 J/cm^2^ and 22.6 to 834.43 J/cm^2^ for 365 and 395 nm LED, respectively (Table 1 and Table 2). The addition of PBS resulted in ~0.06 log reduction in the cell counts due to the dilution effect. The sample in PBS and without the LED treatment was taken as the control for comparison of the effect of power levels on the inactivation efficacy of the LED treatments. The treated and untreated samples were serially diluted in sterile TSBYE (Tryptic Soy Broth with 0.6% Yeast extract) and the viable cell counts were obtained by spread plating on TSAYE (Tryptic Soy agar with 0.6% Yeast Extract) plates and incubating the plates for 20–24 h at 37 °C. The detection limit was 2 log (CFU/mL).

### 2.4. Inactivation of Dry E. coli and Salmonella enterica

Dried and equilibrated cells of *E. coli* AW1.7 and *S. enterica* ATCC13311 (10 mg) with 0.75 a_w_ were spread on a small disk made up of plant-based and biodegradable PLA (Polylactic acid) filament with an area of 7.07 cm^2^. The samples were kept at 4 cm from the LED head and treated at power levels of 60% with 365 and 395 nm LEDs. For *S. enterica* with 365 nm LED, an additional power level of 100% was used. The treatment times chosen were 10, 20, 30, 45 and 60 min for both the LEDs. Sample without LED treatment was taken as control. To determine the effect of varying initial inoculum levels (10^8^, 10^7^ and 10^6^ CFU/cm^2^) of *Salmonella enterica*, the bacteria dried on a coverslip equilibrated to 0.75 a_w_ was used for the treatment with 365 and 395 nm LED with a similar dose of 139 J/cm^2^, corresponding to treatment times of 45 and 10 min, respectively. The equilibrated sample without LED treatment was taken as control. Viable cell counts were determined by surface plating of serial dilutions in 0.1% peptone water as described in Section 2.3.

### 2.5. Inactivation of Salmonella Cells on Low-a_w_ Pet Food Pellets

Four pellets of the equilibrated dry pet foods, weighing 0.6 g were treated with 365 and 395 nm LED with a total dose of ~658 J/cm^2^ in a benchtop humidity chamber (BTL-433, ESPEC North America Inc., Hudsonville, MI, USA). The treatment temperature and relative humidity maintained inside the humidity chamber were 25 °C and 75%, respectively, and the distance between the sample and the LED head was kept at 2 cm. A cooling fan (DC Brushless fan, 50 × 50 × 15 mm, model BB5015H12, HK fans, Shenzen, China) with a voltage of 7 V supplied by a DC power supply (KD3005D, Digital Control DC linear power supply, Korad, Shenzen, China) was placed 2.7 cm away from the samples during the treatment with both the LEDs to reduce the increase in the surface temperature. Re-humidification of the pet foods to a a_w_ of 0.75 at the end of the LED treatment was carried out by incubating the treated samples inside the humidity chamber set to 25 °C and 75% relative humidity for 30 min. To understand the effect of intermittent LED treatments, pellets were treated in two ways, (1) continuous treatment that involved the treatment of samples continuously for a total dose of ~658 J/cm^2^, corresponding to a treatment time of 20 min for 395 nm and 96 min for 365 nm, followed by re-humidification to 0.75 a_w_ and (2) non-continuous treatment, which involved the treatment of the pellets for a dose of ~329 J/cm^2^, corresponding to 10 min for 395 nm and 48 min for 365 nm LED, followed by spraying of 15 µL of autoclaved water on each pellet and immediately followed by another LED treatment for a dose of ~329 J/cm^2^ and a final re-humidification to 0.75 a_w_. The equilibrated pet food pellets without any LED treatment was taken as the control. For enumeration, the treated and non-treated pet foods were homogenized in a stomacher bag with 100 mL of autoclaved 0.1% peptone water by using the stomacher (Seward, London, UK). Then, 100 µL of the homogenized samples was used for serial dilution and enumeration was done, as described in Section 2.3.

### 2.6. Weight Loss, Water Content and Water Sorption Isotherms of the Treated Samples

Weight loss due to evaporation of water during the LED treatments was determined by measuring the weights of samples before and after the treatments. To determine the initial water content of dry *S. enterica* equilibrated to 0.75 a_w_, 1 g of sample was dried in a gravity convection oven (Heratherm OGS60, Thermo Scientific, Waltham, MA, USA) in triplicates at 105 °C for 8 h and the dry weight of the bacteria was determined and the water content (dry-basis) was calculated. Similarly, the water content (dry basis) for pet foods equilibrated to 0.75 a_w_ was analyzed by drying 3.5 g of pet food pellets in triplicates in the convention oven at 105 °C for until constant final weight was achieved. The change in the a_w_ after the treatments were recorded using a water activity meter (4TE, patent number 5816704, Aqualab, Pullman, WA, USA).

The desorption isotherms for *S. enterica* were prepared by using a Vapor Sorption Analyzer (VSA, Meter group, Inc., Pullman, WA, USA) [17,33] at ambient (20 °C) and treatment temperatures corresponding to 365 and 395 nm LED treatments (i.e., 32 and 55 °C, respectively). These treatment temperatures were selected based on the temperature increase observed in the samples during the LED treatments. To develop the isotherms, dry *Salmonella* samples (approximately 0.6 g) were exposed to selected relative humidity values corresponding to the water activities 0.1 to 0.8 inside the VSA. The equilibrium water contents at the selected water activities were determined automatically by monitoring the mass of the samples at equilibrium conditions. To map the drying process of *Salmonella* samples, we used their desorption isotherms to determine their approximate final a_w_, using the water content data. The isotherm modeling was done by using the Guggenheim-Anderson-de Boer (GAB) equation (Equation (1)) as a best fit [34]:

(1) $$\frac{X}{X_{m}}=\frac{{\mathrm{CKa}}_{w}}{{(1-\mathrm{Ka}}_{w}{)(1-\mathrm{Ka}}_{w}{+\mathrm{CKa}}_{w})}$$

where X is the water content (dry-basis), X_m_ is the water content of the monolayer (dry-basis) and C, K and X_m_ are the temperature dependent parameters which can be expressed as Equations (2), (3) and (4), respectively:

(2) $${C\;=\;C}_{0}\exp(\frac{{\Delta H}_{c}}{\mathrm{RT}})$$

(3) $${K\;=\;K}_{0}\exp(\frac{{\Delta H}_{k}}{\mathrm{RT}})$$

(4) $$X_{m}{\;=\;X}_{\mathrm{mo}}\exp(\frac{\Delta H_{x}}{\mathrm{RT}})$$

where R corresponds to gas constant and T is the temperature. In Equation (2), ∆H_c_ is the difference in enthalpy between monolayer and multilayer sorption and is generally positive. In Equation (3), ∆H_k_ is the difference between heat of condensation of water and the heat of sorption of multimolecular layer [17,35].

### 2.7. Statistical Analysis

The experiments were done independently in triplicates (*n* = 3). The statistical analysis was done using the SAS version 5.1.26 (SAS Institute Inc., Cary, NC, USA) and the significance was taken by Tukey’s LSD test (*p* < 0.05). The effect of both the LED treatments on the dry bacterial samples were assessed by three-way ANOVA. Comparison of the effect of different power levels for each LED treatments, the effect of LED treatments on pet foods and the effect of LED treatments on the water loss were analyzed by two-way ANOVA.

## 3. Results

### 3.1. Antibacterial Efficacy of 365 and 395 nm LED Treatments

**Figure 1:**
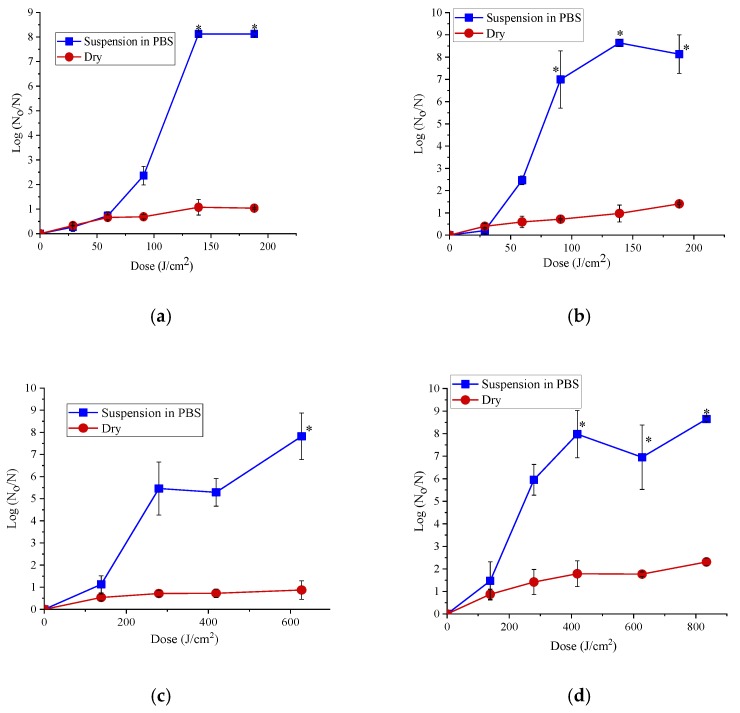
The efficacy of 365 nm LED against *E. coli* (**a**), *Salmonella enterica* (**b**) and the efficacy of 395 nm LED against *E. coli* (**c**) *Salmonella enterica* (**d**) suspended in PBS (phosphate buffered saline, pH 7.4) and dried bacteria for different dose treatments at 60% power level with 4 cm height between the sample and the LED head. The dose used for 365 nm were 28.9 (10 min), 59.2 (20 min), 90.9 (30 min), 139.1 (45 min) and 188.1 (60 min) J/cm^2^. The dose used for 395 nm were 138.8 (10 min), 279.2 (20 min), 418.7 (30 min), 627.3 (45 min) and 834.4 (60 min) J/cm^2^. Here, N_0_ represents the CFU/mL in control and N represents the CFU/mL in the treated samples of bacteria suspended in PBS. For dry bacteria, N_0_ represents the CFU/g in control and N represents the CFU/g in the treated samples. Error bars indicate the standard deviation (*n* = 3). An asterisk (*) indicates the reduction of cell counts below the detection limit.

To compare the effect of LED treatment on high and low-a_w_ *E. coli* and *Salmonella*, the cells were suspended in PBS or equilibrated to a_w_ 0.75 and treated with 365 or 395 nm light pulses. Bacterial cells suspended in PBS were more sensitive than the dry bacteria. The 365 and 395 nm LED treatments produced a reduction of ~8 log CFU/g (below the detection limit) in *E. coli* and *Salmonella enterica* cells suspended in PBS compared to a maximum reduction of ~1 to 2 log CFU/g in dried bacterial cells (Figure 1). Treatments with the dose of 139 J/cm^2^ were performed at both wavelengths, and thus, allowed for direct comparison. The 395 nm LED required higher energy input compared to 365 nm for achieving same microbial inactivation level. For example, for the same dose of 139 J/cm^2^, the 395 nm LED treatment produced 1.13 and 1.46 log reduction compared to 8.12 and 8.63 log reduction with 365 nm LED treatment in *E. coli* and *Salmonella enterica* suspended in PBS, respectively. Similarly, the 365 nm LED showed significantly (*p* < 0.0001) better antibacterial efficacy compared to the 395 nm LED for same dose in the dry *Salmonella enterica* and *E. coli* cells. Dried cells of *E. coli* were moderately more resistant to 395 nm LED treatments than *S. enterica*.

The 365 nm LED treatment produced significantly (*p* < 0.0001) higher antibacterial effect in *E. coli* cells suspended in PBS compared to 395 nm for the same dose. For instance, treatments with ~92 J/cm^2^ reduced cell counts by about 7 log (CFU/mL) after treatments with 365 nm but only by about 1 log (CFU/mL) after treatment with 395 nm. After treatment of dry *Salmonella enterica* cells with 365 or 395 nm, the reduction of cell counts remained less than 1 log (CFU/g) even at an energy input of 139.1 and 138.8 J/cm^2^, respectively (Figure 1) Overall, the dose had major effect on the antibacterial effect of both the LEDs than the pulse width (power levels) of the light produced.

### 3.2. Effect of Power Levels on the Antibacterial Efficacy of the LEDs

**Figure 2:**
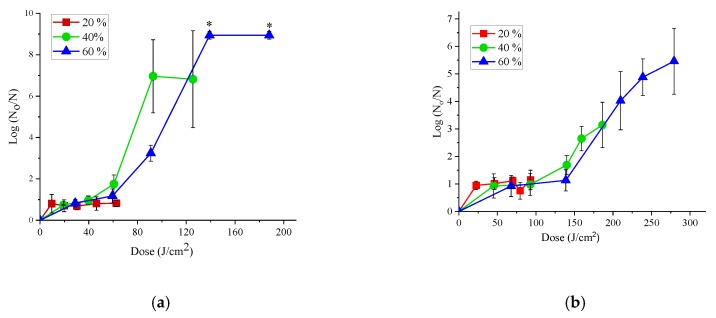
Effect of power levels on the inactivation efficiency of 365 nm (**a**) and 395 nm (**b**) LED against *E. coli* AW1.7 suspended in PBS, subjected to different doses of LED treatment. The doses used for 365 nm LED treatments corresponding to treatment times 10, 20, 30, 45 and 60 min, were 9.6, 19.8, 30.3, 46.3 and 62.6 J/cm^2^ for 20% power level, 19.2, 39.6, 60.6, 92.8 and 125.4 J/cm^2^ for 40% power level and 28.9, 59.2, 90.9, 139.1 and 188.1 J/cm^2^ for 60% power level, respectively. The doses used for 395 nm LED treatments corresponding to treatment times 5, 10, 15, 17 and 20 min were 22.6, 46.2, 69.9, 79.5 and 92.98 J/cm^2^ for 20% power level, 45.2, 92.6, 139.9, 159.2 and 186.2 J/cm^2^ for 40% power level and 67.8, 138.8, 209.9, 238.7, 279.2 J/cm^2^ for 60% power level, respectively. Here, N_0_ represents the CFU/mL in the control and the N represents the CFU/mL in the treated samples. Error bars indicate the standard deviation (*n* = 3). An asterisk (*) indicates the reduction of cell counts below the detection limit.

Increasing the power level of the LED system increased the dose and pulse of the light (Table 1 and Table 2), and hence increased the antibacterial efficacy at higher power levels. To understand the effect of power levels, *E. coli* suspended in PBS were treated with 20, 40 or 60% power levels with 365 or 395 nm LEDs. For low power level of 20%, doses did not produce any significant effect on the inactivation of *E. coli* suspended in PBS for both 365 and 395 nm LEDs. However, increasing the dose above 60 J/cm^2^ at 40% and above 139 J/cm^2^ at 60% power levels resulted in the significant (*p* < 0.0001) effect of dose on the antibacterial efficacy of both 365 and 395 nm LED treatments (Figure 2). For the similar dose of 91 J/cm^2^, treatment with 365 nm LED produced a reduction of 6.96 log (CFU/g) at 40% power level (45 min) compared to 3.24 log (CFU/g) at 60% power level (30 min), indicating that no linear effect of power level was observed and the reduction was not influenced by power setting but by the overall dose or energy input.

### 3.3. Antibacterial Efficacy of LED in Low-a_w_ Pet Foods

**Figure 3:**
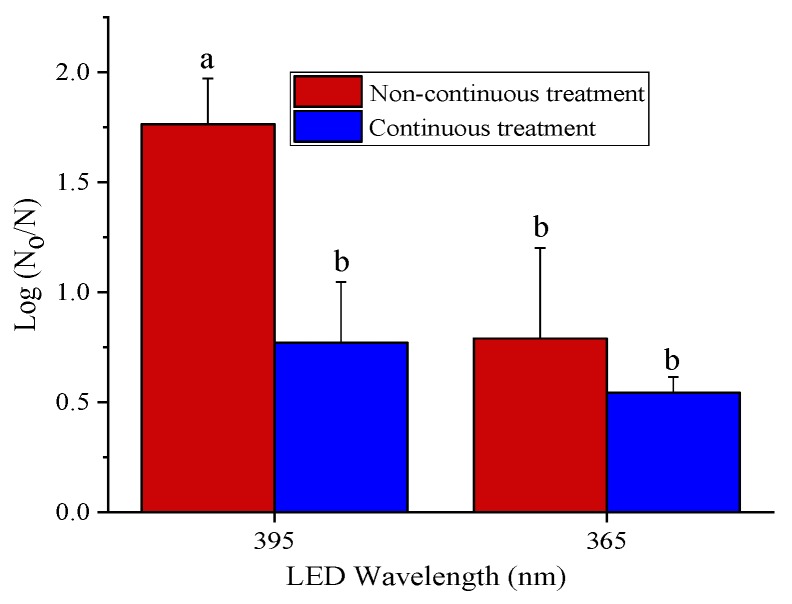
The efficacy of 365 and 395 nm LED against 5 strain cocktail of *Salmonella enterica* spp. in low-a_w_ pet food pellets equilibrated to 0.75 a_w_ when the height between the sample and the LED head was maintained at 2 cm. The non-continuous treatment involved the treatment of the pellets for a dose of ~329 J/cm^2^, corresponding to 10 min for 395 nm and 48 min for 365 nm LED, followed by spraying of 15 µL of autoclaved water on each pellet and immediately followed by another LED treatment for a dose of ~329 J/cm^2^ and a final re-humidification to 0.75 a_w_. The continuous treatment involved the treatment of samples continuously for a total dose of ~658 J/cm^2^, corresponding to a treatment time of 20 min for 395 nm and 96 min for 365 nm, followed by re-humidification to 0.75 a_w_. Here, N_0_ represents the CFU/g in control and the N represents the CFU/g in the treated pet foods. Bars with same letter are not significantly different (*p* < 0.05). Error bars indicate the standard deviation (*n* = 3).

We determined the effectiveness of both 365 and 395 nm LEDs against a *Salmonella enterica* cocktail of five strains inoculated on dry pet foods equilibrated to 0.75 a_w_ treated at the same total dose of ~658 J/cm^2^ in an enclosed humidity chamber set at 25 °C and 75% relative humidity. The 395 nm LED treatment produced significantly (*p* = 0.007) better inactivation in *Salmonella enterica* on pet foods with the non-continuous treatment (method 2) compared to the continuous treatment (method 1), (Figure 3). Overall, 395 nm LED showed better antimicrobial effectiveness on pet foods compared to 365 nm LED, contrary to the trend observed with dry bacteria, indicating that the LED’s antibacterial efficacy was dependent on the product parameters and the strains used for the treatment.

### 3.4. Change in Temperature during LED Treatment

The surface temperature changes were monitored during LED treatments of bacterial samples at high and low-a_w_ conditions. The initial temperature of the untreated high and low-a_w_ bacterial samples was 21–23 °C. The temperature of *E. coli* and *Salmonella enterica* cultures suspended in PBS during 365 nm LED increased to 25–27 °C after treatment with 188.07 J/cm^2^ dose; treatments with 395 nm at 418.7 J/cm^2^ dose increased the temperature to 34–36 °C after 30 min. In dry bacterial samples, we observed a higher surface temperature increase compared to the bacterial suspension in PBS. For example, 365 nm LED treatment with 188.07 J/cm^2^ dose (60 min) increased the temperature of the bacteria to 33 °C while the 395 nm LED treatments increased the temperature to maximum of 53–55 °C with 834.43 J/cm^2^ dose (60 min).

Similarly, in pet foods, a dose of ~329 J/cm^2^ with 365 nm LED treatments increased the temperature to 34–35 °C and a continuous treatment with ~658 J/cm^2^ increased the temperature to 32–35 °C from 25 °C, at 60% power level. However, the 395 nm LED treatment increased the temperature from 25 °C to 57–62 °C when the dose was ~329 J/cm^2^ (10 min) and to 66–67 °C with continuous treatment with ~658 J/cm^2^ (20 min) dose. Overall, a greater surface temperature increase was observed with 395 nm LED treatments compared to 365 nm LED treatments at the same treatment times and power level.

### 3.5. Change in Water Content and Water Activity of Bacterial Samples and Pet Foods during LED Treatments

The LED treatments resulted in weight loss which is associated with water loss due to drying of the samples during both 365 and 395 nm LED treatments. The water content (dry basis) of the dry *Salmonella enterica* cells equilibrated to 0.75 a_w_ was 0.168 kg water/kg dry solids. The 395 nm LED treatments reduced the water content of *Salmonella enterica* more when compared to 365 nm LED treatments. The maximum reduction in water contents after 365 and 395 nm LED treatments were 15.5 and 50.6%, respectively (Table 3).

**Figure 4:**
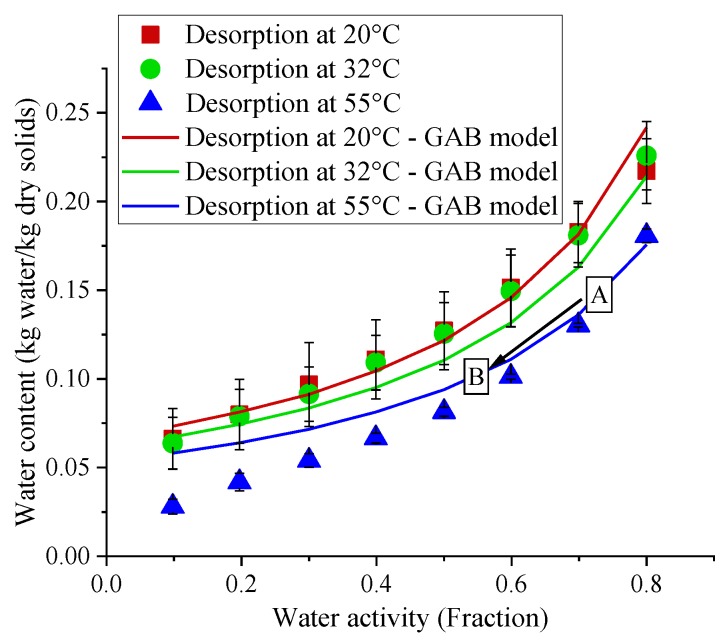
The desorption isotherm of dry *Salmonella enterica* Typhimurium ATCC13311 at 20, 32 and 55 °C. The scatter plots correspond to the desorption isotherm developed by the Vapor Sorption Analyzer (VSA). The line plots represent the desorption isotherms as predicted using the GAB (Guggenheim-Anderson-de Boer) model. Error bars indicate the standard deviation (*n* = 2).

During the 60 min (834.4 J/cm^2^) treatment with 395 nm LED, the suspension of bacteria in PBS were dried up entirely, due to the high light energy dose and the temperature increase during the LED treatments, resulting in the evaporation of water. To determine the extent of drying in dry bacteria, the desorption isotherms of *Salmonella enterica* cells and their water contents obtained from weight loss data after LED treatments were used to determine the final a_w_ values of cells after LED treatments (Figure 4). The isotherm data was fitted with the GAB equation (Equation (1)) and the a_w_ values after the LED treatments were determined using this equation (Table 3). To use the desorption isotherm in a_w_ calculations, we assumed that a pseudo water vapor equilibrium existed at the interface of the bacterial surfaces and the surrounding air during long LED treatments (especially for long treatments, i.e., after 60 min). Even though drying is a dynamic process, we made this important assumption to use the desorption isotherms of samples at ambient and LED treatment temperatures to determine the final a_w_ values of the bacterial samples after LED treatments. The determined a_w_ values of bacterial samples using the desorption isotherms showed that, after 365 and 395 nm LED treatments, the a_w_ values reduced significantly (*p* = 0.0054) (Table 3). For the same dose of 139 J/cm^2^ with 365 and 395 nm LEDs corresponding to 45 and 10 min, the a_w_ were reduced to 0.64 and 0.7, respectively (Table 3). In Figure 4, after 60 min (834.43 J/cm^2^) treatments with the 395 nm LED with 60% power level, the water content and a_w_ values were reduced from 0.168 kg water/kg dry solids and 0.75 (Box A) to 0.1 kg water/kg dry solids and 0.54 (Box B), respectively (see black arrow in Figure 4).

**Figure 5:**
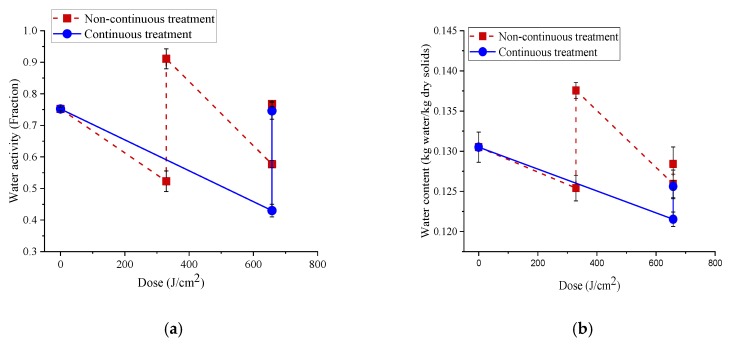

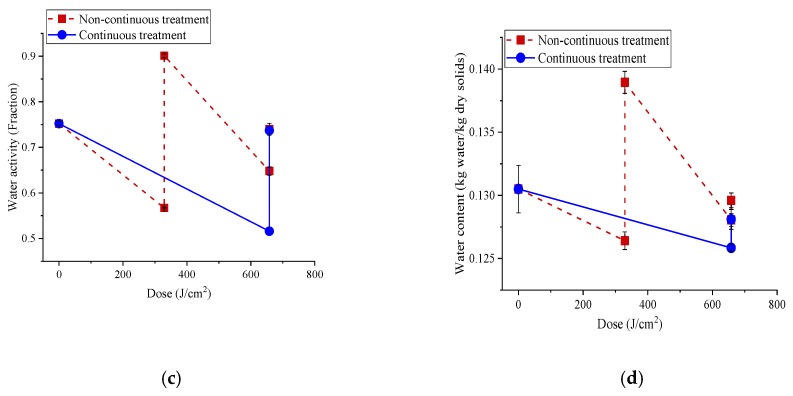
The mapping of a_w_ (**a**) and water content (dry-basis) (**b**) of low-a_w_ pet foods equilibrated to 0.75 a_w_ in both continuous and non-continuous treatments with 395 and a_w_ (**c**) and water content (dry-basis) (**d**) with 365 nm LED. The non-continuous treatment involved the treatment of the pellets for a dose of ~329 J/cm^2^, corresponding to 10 min for 395 nm and 48 min for 365 nm LED, followed by spraying of 15 µL of autoclaved water on each pellet and immediately followed by another LED treatment for a dose of ~329 J/cm^2^ and a final re-humidification to 0.75 a_w_ in the humidity chamber. The continuous treatment involved the treatment of samples continuously for a total dose of ~658 J/cm^2^ corresponding to a treatment time of 20 min for 395 nm and 96 min for 365 nm, followed by re-humidification to 0.75 a_w_ in the humidity chamber. Error bars indicate the standard deviation (*n* = 3).

The initial water content (dry basis) of pet food pellets was obtained as 0.131 kg water/kg dry solids. We determined the changes in their water content and a_w_ during continuous and non-continuous LED treatments (Figure 5). Here, the non-continuous treatment involved sequential LED treatments and re-humidification using water spray. The maximum weight loss observed in the LED treated pet foods was 6–7% in the case of 395 nm treatment and 3.3–4.5% with 365 nm. Consequently, the water content of the pet foods significantly (*p* = 0.001) reduced by 3 and 5.4%, respectively, with continuous and non-continuous treatments after 395 nm LED treatments, while the 365 nm LED treatments reduced the water content by 2 and 3%, respectively (Figure 5). The reduction in the a_w_ of the pet foods was higher in the case of continuous treatment with 395 nm LED treatment compared to 365 nm LED treatment. During the non-continuous treatment, the a_w_ and water contents of pet foods reduced during the first treatment with ~329 J/cm^2^ dose of 365 and 395 nm LEDs, then increased with water spray and again decreased during subsequent LED treatments with a dose of ~329 J/cm^2^. However, the final a_w_ observed after the second LED treatment was higher than the final a_w_ observed after the first LED treatment (Figure 5).

## 4. Discussion

The LED system is cost-effective and its implementation to the existing processing lines is relatively simple, owing to their small size and convenience in using them [36]. In this study, we observed that 365 and 395 nm pulsed LED treatments reduced bacterial cell counts in their suspension in PBS, while the LED treatments were much less effective in inactivating dry cells. This observation confirms the results of another study, where the treatment of *E. coli* suspension with 365 nm LED for 75 min (315 J/cm^2^ dose) reduced cell counts by ~5.7 log CFU/mL: [23]. The energy input and the LED wavelength were the most important factors influencing treatment lethality. The distance of the sample from LED source, dose, treatment time and sample type additionally influence the inactivation efficiency.

Gram negative bacteria such as *E. coli* and *Salmonella enterica* survive and remain infectious in low-a_w_ environments for extended periods of time [37]. The antimicrobial efficacy of 405 nm LED treatment has been extensively reported in relatively high-a_w_ food products, such as fresh cut papaya and ready-to-eat salmon [13,38,39]. Kim et al. [40] studied the inactivation of *E. coli* and *Salmonella enterica* on fresh cut mango by 405 nm LED treatment, where 36 h treatment resulted in cell count reductions of 1 log CFU/cm^2^ with a maximum dose of 3.6 kJ/cm^2^. *E. coli* K12 was susceptible to 395 nm LED in a dose-dependent manner [25]. However, only few studies reported the use of LED treatment in low-a_w_ foods. Better sensitivity of suspension of bacteria in PBS than dry bacteria observed in this study could be attributed to the low penetration capacity of the light emitted by the LED or shadowing of cells in the dry powdered bacteria and the low a_w_ of the cells. Decreasing the inoculum level from 10^8^ to 10^6^ CFU/cm^2^ did not increase in the inactivation of *Salmonella enterica* (data not shown), indicating that a shadowing effect is not critical, or that the non-uniform layer of the bacteria on the boundary of the inoculum had a higher cell density [41].

The maximum log reduction obtained in dry *E. coli* and *Salmonella enterica* was 1.36 and 2.3 log CFU/g, respectively. This low reduction is justifiable in this study as the strains used were already highly resistant and were ideal to study the effect of LED treatment in dry conditions. These strains were subjected to further stress as the bacterial cells were dried before their equilibration to 0.75 a_w_. Under stress, accumulation of trehalose, heat shock proteins, etc. might occur as part of the adaptation mechanism of the bacterial cells, which might result in the increased resistance of these cells to further stress conditions or antimicrobial treatments [4].

We observed that 365 and 395 nm LEDs were effective in the inactivation of *Salmonella enterica* cocktail in pet food pellets. However, higher dose treatments were required, probably due to the collective resistance of five strains of *Salmonella*, *enterica* compared to a single strain tested in the study with the dry bacteria. *Salmonella enterica* FUA1946, FUA1934, FUA1955, especially, were determined as highly resistant to thermal treatment and high pressure CO_2_ from our recent studies (data not shown). Additionally, surface characteristics such as roughness and the composition of pet foods might have interfered with the LED treatment and may have contributed to the increased resistance of *Salmonella enterica* on the pellets. In food systems, higher energy may be generally required to achieve the same level of reduction in comparison to pure microbial cells [42,43]. Similarly, the 405 nm LED treatment on *Salmonella enterica* cocktail inoculated almonds (a low-a_w_ food) produced 0.49 to 0.64 log reduction in almonds [44]. We observed that the non-continuous treatment of pet foods led to better inactivation with 395 nm LED (Figure 3). Addition of water increased the susceptibility of the bacterial cells to the LED treatment and probably aided in more production of reactive oxygen species (ROS) than continuous treatment. Additionally, changes in the a_w_ of the pet foods during non-continuous treatment might have produced an osmotic stress on the cells. The dehydrated cells during the LED treatment might undergo increased membrane permeabilization and cell shrinkage followed by formation of membrane vesicles, previously observed in *E. coli* [45]. Moreover, rehydration of the cells might not help the cells to recover from this permeabilization and changes in membrane functionality and integrity. The sudden dehydration and rehydration might also affect the cell viability [45,46], causing further inactivation in the bacterial cells. Similarly, other decontamination methods that involve subjecting raw almonds to high pressures in water followed by drying at high temperatures resulted in the reduction of *Salmonella enterica* to undetectable levels [47]. Overall, the 365 nm LED treatments were better than 395 nm LED treatments for dry bacteria but the trend was opposite for their inactivation in the pet foods, and this observation deserves further research.

The 365 nm LED treatments showed a smaller increase in the temperature of liquid suspension or dry bacteria compared to 395 nm LED treatments. Similarly, an increase in the surface temperature was observed with 405 nm LED treatment of fresh-cut papaya [38]. The greater temperature increases in the case of 395 nm compared to 365 nm LED treatments could be due to its higher intensity at same power levels and treatment times, which resulted in an increased drying rate in bacterial samples. The temperature increase during LED treatment might have contributed to the inactivation observed in addition to the effect of light emitted. We also observed drying with reduction in weight and water content of the dry *Salmonella enterica* samples after both the LED treatments. The higher intensity of 395 nm increased the drying rate and water loss. The inactivation efficacy of the high intensity pulsed LED could be due to the drying observed as well.

We conducted LED treatments in open laboratory conditions, which resulted in the drying of samples and loss of light energy to the surroundings. Therefore, preventing the loss of LED light during the treatments might improve the antibacterial effect of the LED. Moreover, the limited penetration of the light in the solid matrix as well as the intrinsic resistance of dry cells resulted in a relatively low reduction of cell counts. The high intensity pulsed LEDs probably work better when combined with an intermediate drying or rehydration step. Therefore, the antibacterial effectiveness of LED could be further improved by the addition of water in dry food as an intermediate step indicating the probability of improving the decontamination efficacy of LED when used with some other technology while maintaining the food quality. Development of such antibacterial technology is important to deal with the highly resistant microorganisms in food that can cause illnesses even when present in small numbers.

## 5. Conclusions

In this study, we observed that 365 and 395 nm LED treatments reduced *E. coli* and *Salmonella enterica* populations significantly at high and low-a_w_ conditions. LED treatments showed antimicrobial effect in pet foods at 0.75 a_w_, based on the treatment conditions and the dose used. The 395 nm LED had a higher irradiance compared to 365 nm LED treatments, which resulted in faster reduction in bacterial population for the same treatment times. The antibacterial efficacy of both the LEDs varied significantly. There was a variation in the susceptibility of the bacteria towards the LED treatments, based on the strain and the condition (liquid suspension or dry powder) of the bacteria. Water loss and reduction in the a_w_ were observed during the LED treatments, indicating the drying of the treated bacterial samples, which was confirmed from the desorption isotherm of bacterial cells. This study showed the potential application of the 365 and 395 nm LEDs as an antibacterial technology to reduce foodborne pathogen population in high and low-a_w_ conditions.

## Figures and Tables

**Table 1:** The total dose (J/cm^2^) reached by 365 nm Light Emitting Diode (LED) for different treatment times at selected power levels of 20, 40, 60 and 100% used in this study at 4 cm height between the sample and the LED source.

Treatment Time (min)	Power Levels			
	20% *	40%	60%	100%
10	9.6	19.2	28.9	48.1
20	19.8	39.6	59.2	99.0
30	30.3	60.6	90.9	151.5
45	46.3	92.8	139.1	231.8
60	62.6	125.4	188.1	313.4

* A power level of 20, 40 and 60% was adjusted by treatment with 2, 4 and 6 ms, respectively, at a frequency of 100 Hz; 100% corresponds to exposure to continuous light.

**Table 2:** The total dose (J/cm^2^) reached by 395 nm LED for different treatment times at selected power levels of 20, 40 and 60% used in this study at 4 cm height between the sample and the LED source.

Treatment Time (min)	Power Levels		
	20%	40%	60%
5	22.6	45.2	67.8
10	46.2	92.6	138.8
15	69.9	139.9	209.9
17	79.5	159.2	238.7
20	92.9	186.2	279.2
30	139.4	279.3	418.7
45	208.9	418.4	627.3
60	277.9	556.6	834.4

**Table 3:** The water content of the dry *Salmonella enterica* Typhimurium ATCC13311 after the 395 and 365 nm LED treatments at 60% power level and the water activity from desorption isotherm.

Treatment Time (min)	Water Content (kg Water/kg Dry Solids) with 395 nm LED	Water Activity Values Determined from Desorption Isotherm with 395 nm LED	Water Content (kg Water/kg Dry Solids) with 365 nm LED	Water Activity Values Determined from Desorption Isotherm with 365 nm LED
0	0.168 ± 0.0124 ^a^	0.74 ± 0.0133 ^a^	0.168 ± 0.0124 ^a^	0.74 ± 0.0133 ^a^
10	0.137 ± 0.0171 ^ab^	0.698 ± 0.2115 ^a^	0.154 ± 0.0069 ^ab^	0.675 ± 0.0204 ^bc^
20	0.113 ± 0.0243 ^bc^	0.594 ± 0.1058 ^ab^	0.155 ± 0.0093 ^ab^	0.678 ± 0.0254 ^bc^
30	0.131 ± 0.0162 ^abc^	0.677 ± 0.0574 ^a^	0.158 ± 0.0042 ^ab^	0.686 ±0.0113 ^b^
45	0.083 ± 0.0267 ^c^	0.374 ± 0.0566 ^b^	0.142 ± 0.0053 ^b^	0.637 ± 0.0184 ^c^
60	0.101 ± 0.0528 ^bc^	0.545 ± 0.2377 ^ab^	0.154 ± 0.0146 ^ab^	0.675 ± 0.0431 ^bc^

Values are given as mean ± standard deviation (*n* = 3). Values in each column with a same letter are not significantly different (*p* < 0.05).
